# The Hidden Dangers of Diagnostic Overshadowing in Psychiatric Patients: A Case Study

**DOI:** 10.1192/j.eurpsy.2025.1508

**Published:** 2025-08-26

**Authors:** I. C. Mandras, A. L. Comsa, M. M. Manea

**Affiliations:** 1 Clinica Psihiatrie 3, Spitalul Județean de Urgență Cluj, Cluj-Napoca, Romania

## Abstract

**Introduction:**

Diagnostic overshadowing, or the tendency to attribute physical symptoms to mental illness, poses a significant risk to psychiatric patients, significantly delaying diagnostic and treatment possibilities. This case study highlights the potentially life-threatening consequences of dismissing physical complaints in patients with a history of mental health disorders.

**Objectives:**

To examine the impact of diagnostic overshadowing on patient care and outcomes, emphasizing the need for comprehensive, unbiased medical assessment, regardless of psychiatric history.

**Methods:**

We present the case of a 72-year-old female patient with an extensive history of multiple psychiatric admissions, primarily for somatization and depressive episodes. The patient’s journey began with an initial admission in our psychiatric service. However, her condition rapidly degenerated, as she developed chest pain, leg numbness, and digestive issues. These symptoms were initially attributed to her psychiatric conditions by the internal medicine team, leading to a critical delay in appropriate medical intervention.

**Results:**

As a consequence, the patient’s condition deteriorated rapidly, culminating in a severe septic state. Further investigation revealed that the sepsis had a pulmonary origin, with Serratia marcescens identified as the causative pathogen. This underscores the potential for seemingly benign symptoms to mask serious underlying infections in vulnerable populations. The patient’s case was further complicated by the emergence of several severe medical conditions, including toxic hepatitis, cardiomyopathy, and valvular insufficiencies, highlighting the potential for cascading health issues when initial symptoms are not thoroughly investigated. In the course of treatment, the patient experienced additional complications arising from medical interventions, most notably drug-induced hepatotoxicity, serving as a reminder of the delicate balance required in managing complex cases and the potential for treatment-related adverse events to further complicate patient care.

**Conclusions:**

This case study underscores the critical importance of conducting thorough and unbiased medical evaluations in psychiatric settings, or in cases where psychiatric history is present. It vividly demonstrates how preconceived notions and unconscious biases regarding psychiatric patients can lead to delayed diagnosis and treatment of serious medical conditions, potentially resulting in life-threatening consequences. The case serves as a wake-up call for healthcare providers to approach each patient with an open mind, regardless of their psychiatric comorbidities.

**Disclosure of Interest:**

None Declared

